# Investigating Immunization With Nucleotide Enzymes of *Schistosoma mansoni*: Nucleoside Diphosphate Kinase and Adenylosuccinate Lyase as New Antigenic Targets Against Schistosomiasis

**DOI:** 10.3389/fimmu.2020.569988

**Published:** 2020-09-23

**Authors:** Túlio di Orlando Cagnazzo, Camila Tita Nogueira, Cynthia Aparecida de Castro, Débora Meira Neris, Ana Carolina Maragno Fattori, Ricardo de Oliveira Correia, Yulli Roxenne Albuquerque, Bruna Dias de Lima Fragelli, Tiago Manuel Fernandes Mendes, Silmara Marques Allegretti, Edson Garcia Soares, Larissa Romanello, Juliana Roberta Torini, Humberto D’Muniz Pereira, Fernanda de Freitas Anibal

**Affiliations:** ^1^Laboratório de Inflamação e Doenças Infecciosas, Departamento de Morfologia e Patologia, Universidade Federal de São Carlos – UFSCar, São Carlos, Brazil; ^2^Departamento de Biologia Animal, Instituto de Biologia, Universidade Estadual de Campinas, Campinas, Brazil; ^3^Laboratório de Citopatologia, Departamento de Patologia e Medicina Legal, Universidade de São Paulo, Ribeirão Preto, Brazil; ^4^Laboratório de Biologia Estrutural, Instituto de Física de São Carlos, Universidade de São Paulo, São Carlos, Brazil

**Keywords:** schistosomiasis, *Schistosoma mansoni*, Nucleoside Diphosphate Kinase, Adenylosuccinate Lyase, immunization

## Abstract

Schistosomiasis, caused by *Schistosoma mansoni* trematode worm, affects more than 1.5 million people in Brazil. The current treatment consists in the administration of Praziquantel, the only medicine used for treatment for more than 40 years. Some of the limitations of this drug consist in its inactivity against schistosomula and parasite eggs, the appearance of resistant strains and non-prevention against reinfection. Thus, the objective of this study was to evaluate the effect of immunization with recombinant functional enzymes of the purine salvage pathway of *S. mansoni*, Nucleoside Diphosphate Kinase (NDPK) and Adenylosuccinate Lyase (ADSL), to evaluate the host immune response, as well as the parasite load after vaccination. For this, Balb/c mice were divided into 5 groups: control (uninfected and untreated), non-immunized/infected, NDPK infected, ADSL infected, and NDPK + ADSL infected. Immunized groups received three enzyme dosages, with a 15-day interval between each dose, and after 15 days of the last application the animals were infected with 80 cercariae of *S. mansoni*. On the 47th day after the infection, fecal eggs were counted and, on the 48th day after the infection, the evaluation of leukocyte response, parasite load, antibody production, cytokines quantification, and histopathological analysis were performed. The results showed that immunizations with NDPK, ADSL or NDPK + ADSL promoted a discreet reduction in eosinophil counts in lavage of peritoneal cavity. All immunized animals showed increased production and secretion of IgG1, IgG2a, and IgE antibodies. Increased production of IL-4 was observed in the group immunized with the combination of both enzymes (NDPK + ADSL). In addition, in all immunized groups there were reductions in egg counts in the liver and intestine, such as reductions in liver granulomas. Thus, we suggest that immunizations with these enzymes could contribute to the reduction of schistosomiasis transmission, besides being important in immunopathogenesis control of the disease.

## Introduction

According to the World Health Organization (WHO), a group of diseases called Neglected Tropical Diseases (NTD) affects approximately 1 billion people in regions with high rates of tropical and subtropical climate poverty, and costs developing countries’ economies billions of dollars every year (1). An important highlight within this group of diseases is schistosomiasis, which affects over 200 million people worldwide, with an estimated at-risk population of 700 million (2). Five species of the schistosoma-genus trematode worm are capable of infecting humans; in Brazil, the species *Schistosoma mansoni* is present, causing the well-known mansonic schistosomiasis. With approximately 1.5 million people living in areas of risk for contamination by the parasite (3), mansonic schistosomiasis represents great importance in socioeconomic terms and impact on public health in the country, since influences from the cognitive response of school-age children to the economic production of the country and its consequent development.

The strategy preconized by WHO (4) for schistosomiasis control aims to prevent morbidity in later life through regular treatment with Praziquantel (PZQ), which is currently the only recommended drug for treatment of the species of schistosome infecting humans. The main control strategy is the mass administration of the drug, however, data from the institution itself show that the population that is at risk of acquiring the disease is not fully achieved (4). Added to this, the fact that the drug is not effective against schistosomula or eggs of *S. mansoni* makes its use restricted (5). Another limiting factor for the indiscriminate use of the drug is the emergence of resistant strains over the years (6). Finally, one of the most important aspects of drug failure is its use as a control method, since it does not prevent reinfection (5, 7, 8).

Due to the inadequacies and limitations of the approaches to control schistosomiasis centered on treatment with PZQ, it is necessary to develop a vaccine for this parasitosis (9). The immune response to *S. mansoni* infection has been extensively studied with the objective of identifying antigens that can elucidate the protective response in immunized individuals. Although there are no vaccines available for human use against schistosomiasis, a study with potential candidates in the clinical phase and in experimental models supports the feasibility of developing an effective vaccine (9).

To characterize new targets for vaccine development, we decided to perform a pre-clinical study using the Nucleosides Diphosphate Kinase (NDPK) and Adenylosuccinate Lyase (ADSL) enzymes. These enzymes are involved in the purine rescue pathway of *S. mansoni*. The parasite does not have the purine synthesis pathway, therefore, the purine rescue pathway is the only way to obtain these molecule (10). The biosynthesis of puric and pyrimidic bases is one of the main pathways studied for the development of drugs and vaccines, because they are directly related to the maintenance of DNA and RNA synthesis (11). Besides that, studies have been using these recombinant enzymes to identify new therapeutic targets (12, 13). Such works show that the enzymes of the purine rescue pathway seem to modulate the infection by *Schistosoma* sp. in different species, but this remains an unclear mechanism.

In this pathway, NDPK enzyme, in addition to being very active, is responsible for converting nucleotide diphosphate into triphosphates, while the enzyme ADSL is responsible for the cleavage of adenylosuccinate to adenosine 5′-monophosphate and fumarat (14, 15). In addition, another possible action of NDPK is to aid in the digestion of the host’s blood, since this protein was found in regurgitation and in the anterior esophagus of adult worms coming into direct contact with the host’s blood (15, 16). There is little information on ADSL in *S. mansoni*, but some studies suggest this is a potential chemotherapeutic target (17, 18). In humans, this enzyme can act in the two purine pathways; on the other hand, in *S. mansoni* it is involved only in the purine rescue pathway. This fact may have caused differences in the enzyme structure between the two species, thus enabling the worm’s ADSL to be a candidate for the vaccine or a target for drugs against schistosomiasis (17). On the other hand, studies with enzymes from the purine rescue pathway as candidates for vaccine against *S. mansoni* are scarce. However, Neris et al. (19) demonstrated an increase in the specific immune response after immunization with enzymes from the *S. mansoni* purine rescue pathway (PNP1, HGPRT, and ADK1).

Therefore, the need for new candidates for vaccines and the influence of the essential metabolic pathways of *S. mansoni* on the survival of the parasite motivated the performance of the present study. The vaccine formulation using the recombinant nucleoid enzymes NDPK and ADSL from the route of purines salvation of *S. mansoni* aimed to evaluate the immunological response developed against the parasite, in addition to improving understanding about infection and, consequently, better understanding about the control and prevention of mansonic schistosomiasis.

## Methodology

### Recombinant Enzymes of *S. mansoni*

The recombinant enzymes of *S. mansoni* (ADSL – code Smp_038030) and (NDPK – code Smp_092750) were produced by insertion of plasmids into bacterial cultures using the protein expression methodology and purified by the affinity chromatography method at the Crystallography Laboratory in the Institute of Physics of São Carlos (IFSC – USP) as previously described (15, 17).

### Animals

Female Balb / c mice, weighing between 15 and 18 *g*, with 4 to 6 weeks of age, were used, from the Animal House Unit II from the Faculty of Pharmaceutical Sciences of Ribeirão Preto, University of São Paulo (FCFRP-USP). The animals have the SPF certificate and the entire experimental design was based on the recommendations of the Ethical Principles for Animal Experimentation and was authorized by the Ethics Committee on the Use of Animals (CEUA) of the Federal University of São Carlos – UFSCar, under the protocol number 2-022/2014.

### Immunization

**FIGURE 1:**
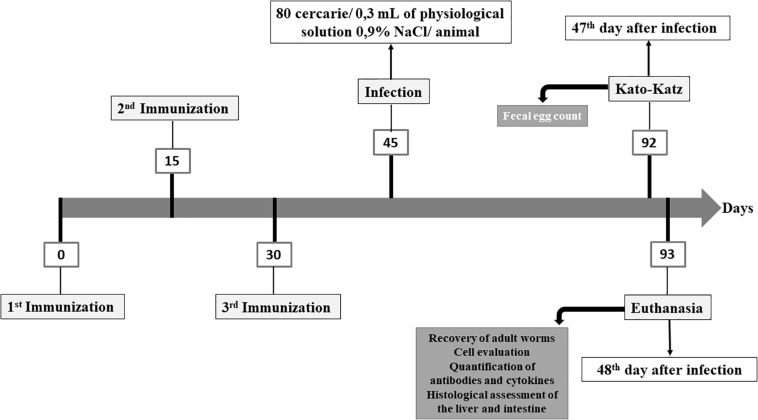
Experimental design for immunization of the animals with the recombinant proteins NDPK and ADSL.

Following the experimental design shown in Figure 1, two independent experiments were carried out with *n* = 6–7 animals/group/experiment. The animals were divided into the following experimental groups: (1) Control group (CTRL): not immunized and not infected; (2) Infected group (INF): not immunized and infected with *S. mansoni*; (3) NDPK group: immunized with the NDPK enzyme and later infected with *S. mansoni*; (4) ADSL group: immunized with the ADSL enzyme and later infected with *S. mansoni* and (5) NDPK + ADSL group: immunized with the mix of enzymes NDPK + ADSL and later infected with *S. mansoni*. The immunization was performed with the application of three doses, with an interval of 15 days between doses. All immunizations were performed intraperitoneally. The groups NDPK and ADSL were immunized with 100 μg of the enzyme, NDPK, and ADSL, respectively, with 100 μg of the adjuvant aluminum hydroxide, solubilized in 1x PBS, totalling 200 μL of final solution per animal. The NDPK + ADSL group was immunized with 100 μg of enzyme (50 μg of NDPK + 50 μg of ADSL), with 100 μg of the adjuvant aluminum hydroxide, solubilized in PBS 1x, totalling 200 μL of final solution per animal.

### Infection of Animals With *S. mansoni*

After 15 days of the last immunization, the animals of the groups INF, NDPK, ADSL, and NDPK + ADSL were challenged with 80 cercariae of *S. mansoni* per animal. Infectious larvae (cercariae) from Belo Horizonte strain (Minas Gerais – Brazil) maintained in the Department of Animal Biology from the Institute of Biology (IB) of the State University of Campinas – UNICAMP, Campinas – SP were used. The procedure was performed by caudal immersion in order to mimic the natural infection promoted by the parasite, as previously described (20).

### Evaluation of Parasitic Load

#### Fecal Egg Count and Adult Worms’ Recovery

Fecal egg count was performed using the Kato-Katz method (21), where the kit used was HELM TEST – Bio-Manguinhos, Fundação Oswaldo Cruz – FIOCRUZ.

The feces of each animal were individually sieved in a filter and mounted on microscopic slides, with a standardized amount of feces through a hole with a known diameter in the plate and covered with cellophane paper impregnated with malachite green, aiming to the conservation of feces and the clearing of eggs of *S. mansoni*. Subsequently, the eggs were counted and the number of eggs per gram of feces was calculated according to the following standardized formula by the kit: number of eggs in the sample per gram of feces = number of eggs found in the slide × factor 24.

Adult worms were recovered through perfusion of the portal system and intestinal mesentery (22). Percentage (%) reduction of the parasitic load was calculated comparing the average of worms recovered from each experimental group and the respective control, according to the following formula (23):

$$\%\text{RPL}=\frac{\text{RCG}-\mathrm{REG}}{\text{RCG}}\times 100$$

where %RPL is the Reduction in the Parasitic Load, RCG is Recovery in the Control Group and REG is Recovery in the Experimental Group.

### Immunological Profile: Eosinophils

#### Peritoneal Cavity Lavage and Blood

The eosinophils from the peritoneal cavity lavage (PCL) and blood were analyzed by extracting tissues from animals in all experimental groups on the 48th day after infection. The animals’ blood was obtained by puncture of the left brachial vein using Ethylenediamine Tetra-acetic Acid (EDTA – from Ethylenediamine tetra acetic acid) as an anticoagulant. To perform the LCP, 3 mL of 1x PBS, pH 7.4, containing 0.5% sodium citrate (citrated PBS) were used. The solution was injected into the peritoneum with a syringe and needle, the region was homogenized, and the cells of the peritoneal region were subsequently recovered. The total number of eosinophils/mm^2^ in both compartments (blood and PCL) were determined using Turk’s solution. at 1:20 dilution. Each sample was counted in a Neubauer chamber. Blood smears were used for the differential counting of blood cells and slides were made in cytocentrifuge for the differential counting of cells of the PCL. Blood and PCL slides were stained using the Rapid Panoptic dye and 100 cells were counted, being differentiated into eosinophils, by optical microscopy, with an increase of 1000.

### Immunoenzymatic Assay

Antibodies and cytokines were investigated from the animals’ total plasma pool by ELISA immunoenzymatic assay (Enzyme Linked Immuno Sorbent Assay), following manufacturer’s instructions for IgG1 (anti-Mouse IgG1 Antibody HRP Conjugated, Bethyl Laboratories, Inc.), IgG2a, IgE, IL-4, and IFN-γ kits (Kit OptEIA^TM^, BD Biosciences), briefly described: in 96-well microtiter plates, 2 μg/well of the enzymes (NDPK, ADSL, and NDPK+ADSL) were applied for sensitization to IgE and IgG2a and 12 μg/well to IgG1 diluted in 0.1 M carbonate buffer – pH 9.5, totalling 100 μL/well, for 16 h at 4±C. For the IL-4 and IFN-γ cytokines, sensitization was performed with the respective primary monoclonal antibody diluted in 0.1 M carbonate buffer – pH 9.5, totalling 100 μL/well, for 16 h at 4±C. After sensitization, the plates were washed with 300 μL/well of 1x PBS + 0.05% Tween 20, pH 7.4 (washing solution). After washing, 200 μL/well of blocking solution (PBS 1x + BSA – Bovine Serum Albumin 1%) were added and the plates were incubated for 1 h. After this period, the plates were washed again. Before applying the samples to the plates, the animals’ plasma was divided into pools of 2 individuals from each experimental group, for each experiment. The samples were applied and incubated for 2 h. The samples were diluted in 1:10 carbonate buffer for the of antibodies analysis (100 μL/well) and for the cytokines were used pure samples (50 μL/well). After the incubation period, the plates were washed. The secondary antibody conjugated with peroxidase enzyme was added, diluted in PBS 1x + 1% BSA, in different proportions for each antibody and cytokine according to the manufacturer and 100 μL/well was added. The plates were then incubated for 1 h and 30 min in the dark. After this period, the plates were washed and 100 μL/well of the TMB substrate (3.3 ’, 5.5’ – Tetramethylbenzidine) and the plates were incubated, still protected from light, by approximately 30 min. Then, the reaction was blocked with the application of 50 μL/well of 2N sulfuric acid. The plates were read at a wavelength of 450 nm by the ELISA plate reader.

### Histology of Liver and Intestines

Liver and intestines were collected 48 days after infection and fixed in buffered formaldehyde. The samples were embedded in paraffin blocks, sectioned in 5 μm sections and stained with Haematoxylin-Eosin (H.E.) and Masson’s Trichrome. The slides were prepared at the Laboratory of Cytopathology, Department of Pathology and Legal Medicine, Faculty of Medicine of Ribeirão Preto – FMRP – USP. The slides were scanned at the 3DHistech Panoramic Desk in the Applied Immunology Laboratory, Department of Genetics and Evolution – DGE, at the Federal University of São Carlos – UFSCar. The images were made using 3DHistech’s Pannoramic Viewer 1.15.4 program.

### Statistical Analysis

The results were expressed as mean ± standard deviation (SD) and analyzed using GraphPad Prism 7.0 (San Diego, CA, United States). Shapiro–Wilk test was used to assess normality. The ANOVA test (unidirectional analysis of variance) was applied to the parametric data and the post-test was performed using the Tukey multiple comparison test. For non-parametric data, the Kruskal–Wallis test was used, and the post-test was performed using Dunn’s multiple comparison test. The statistical significance considered was *p* < 0.05.

## Results

### Evaluation of the Parasitic Load

**FIGURE 2:**
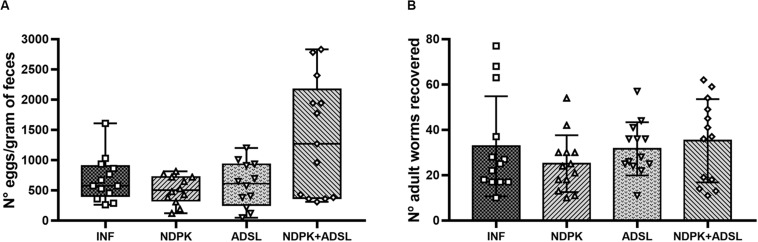
Number of eggs in feces **(A)** and adult worms recovered from the hepatic portal system **(B)** from two independent experiments (*n* = 6–7 animals/group/experiment). The numbers of eggs/gram of feces data represent the first and third quartiles at the top and bottom of the box plot graph, the middle line represents the median value and the minimum and maximum values are represented by the error bars (whiskers). The numbers of adult worms recovered data represent the mean ± SD. There was no statistical difference between the results of the immunized groups when compared with the INF group. The geometric figures represent the dispersion of the data for each group.

Figure 2 represents the number of eggs in feces and worms recovered from the mice’s hepatic vein. There was no statistical difference between the immunized groups and the INF group. The INF immunized with both recombinant enzymes (NDPK + ADSL), showed the highest values when compared to the other groups, in addition to the data of eggs/feces found in each animal being more dispersed in this group.

### Quantification of Eosinophils in the Blood and in the Peritoneal Cavity Lavage

**FIGURE 3:**
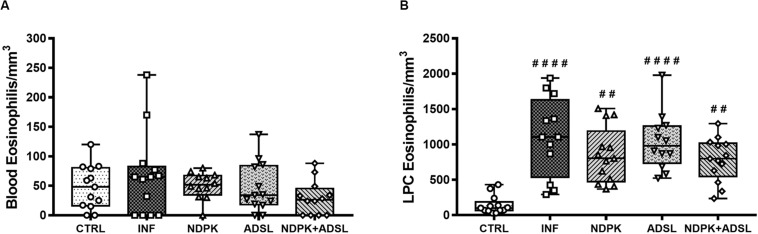
Number of eosinophils in the blood **(A)** and in the PCL **(B)** 48 days after infection with *S. mansoni* and 93 days after the first immunization. The upper and lower part of the Box plot graph represents the first and third quartiles, the middle line represents the median value and the minimum and maximum values are represented by the error bars (whiskers). (#) Represents a statistically significant difference from two independent experiments (*n* = 6–7 animals) using the Kruskal–Wallis non-parametric test followed by Dunn’s post-test between the results of the experimental groups when compared with the CTRL group; ##*p* < 0.01; ####*p* < 0.0001. The geometric figures represent the dispersion of the data for each group.

The Figure 3 represents the number of eosinophils in the blood and PCL of animals in the CTRL group and animals infected and immunized or not with NDPK, ADSL, and the association between NDPK and ADSL. In the blood (Figure 3A), there was no statistically significant difference in the number of eosinophils between the groups. In the PCL (Figure 3B) there was a significant increase in the number of eosinophils in all groups when compared to the CTRL group. However, the number of eosinophils in all immunized groups was lower than that of the INF group, but without statistical significance.

### Quantification of Cytokines in Plasma

**FIGURE 4:**
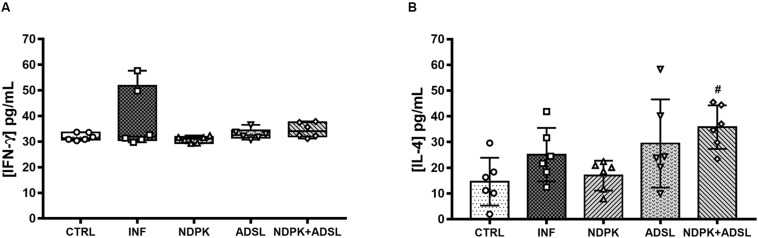
Concentrations of IFN-γ **(A)** and IL-4 **(B)** cytokines in plasma pool 48 days after infection with *S. mansoni* and 93 days after the first immunization of two independent experiments (*n* = 6–7 animals). The IFN-γ cytokine data represent the first and third quartiles at the top and bottom of the box plot graph, the middle line represents the median value and the minimum and maximum values are represented by the error bars (whiskers). The IL-4 cytokine data represent the mean ± SD. (#) represents statistically significant difference using the One-Way ANOVA parametric test followed by the Tukey’s post-test between the results of the experimental groups when compared with the CTRL group; ^#^*p* < 0.05. The geometric figures represent the dispersion of the data for each group.

The concentrations of IFN-γ and IL-4 present in the animals’ plasma pool are shown in Figure 4. IFN-γ did not differ statistically between groups, despite being more present in the INF group (Figure 4A). The plasma concentrations of IL-4 were higher in the NDPK + ADSL group when compared to the CTRL group (Figure 4B).

### Detection of Antibodies Present in Plasma

**FIGURE 5:**
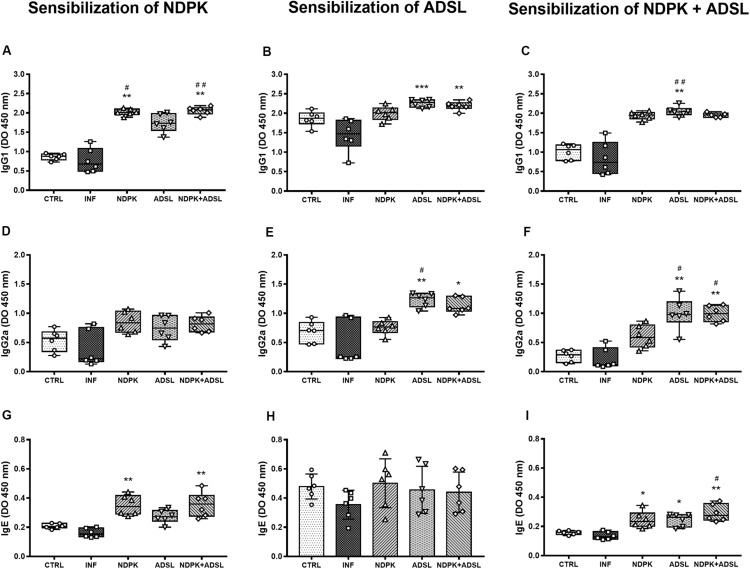
Detection of IgG1 **(A–C)**, IgG2a **(D–F)**, and IgE **(G–I)** antibodies in a plate sensitized with NDPK, ADSL, and NDPK+ADSL in the plasma pool of mice 48 days after infection with S. *mansoni*, and 93 days after the first immunization of two independent experiments (*n* = 6–7 animals). Nonparametric data were analyzed using the Kruskal–Wallis test followed by Dunn’s post-test and are represented in Box plot, with the lower and upper lines being the first and third quartiles, the middle line being the median value and the minimum and maximum values are represented by error bars (whiskers). Parametric data were analyzed by the One-Way ANOVA test followed by the Tukey’s post-test and are represented in the bar graph through the mean ± SD. (#) represents a statistically significant difference between the results of the experimental groups when compared with the CTRL group; ^#^*p* < 0.05; ^##^*p* < 0.01. (*) It represents a statistically significant difference between the results of the experimental groups when compared with the INF group; *p < 0.05; **p < 0.01; ***p < 0.001. The geometric figures represent the dispersion of the data for each group.

The evaluation of the production of IgG1, IgG2a, and IgE antibodies in a plate sensitized with recombinant NDPK protein from *S. mansoni*, caused a significant increase in IgG1 production in the INFs, NDPK, ADSL, and NDPK + ADSL immunized, compared to the group CTRL and INF (Figure 5A). In the production of IgE, the behavior was similar in that, only the groups NDPK and NDPK + ADSL had higher values compared to the group CTRL and INF (Figure 5G), but IgG2a showed no difference between the groups (Figure 5D).

Considering the levels of IgG1, IgG2a, and IgE antibodies in a plate sensitized with the recombinant enzyme ADSL from *S. mansoni*, there was an increase in the production of IgG1 in the ADSL and NDPK + ADSL groups compared to the INF (Figure 5B). In the production of IgG2a, the ADSL, and NDPK + ADSL groups also showed higher values when compared to the INF (Figure 4E). IgE production did not differ between groups (Figure 5F). When there was sensitization with the recombinant protein NDPK + ADSL from *S. mansoni*, a significant increase in IgG1 production was observed in the group immunized with ADSL compared to the IFN group (Figure 5C). IgG2a was higher in the ADSL and NDPK + ADSL groups compared to the IFN and CTRL controls. IgE production was higher in the three immunized groups (NDPK, ADSL, and NDPK + ADSL) compared to INF (Figure 5I).

### Histopathology of the Liver

**FIGURE 6:**
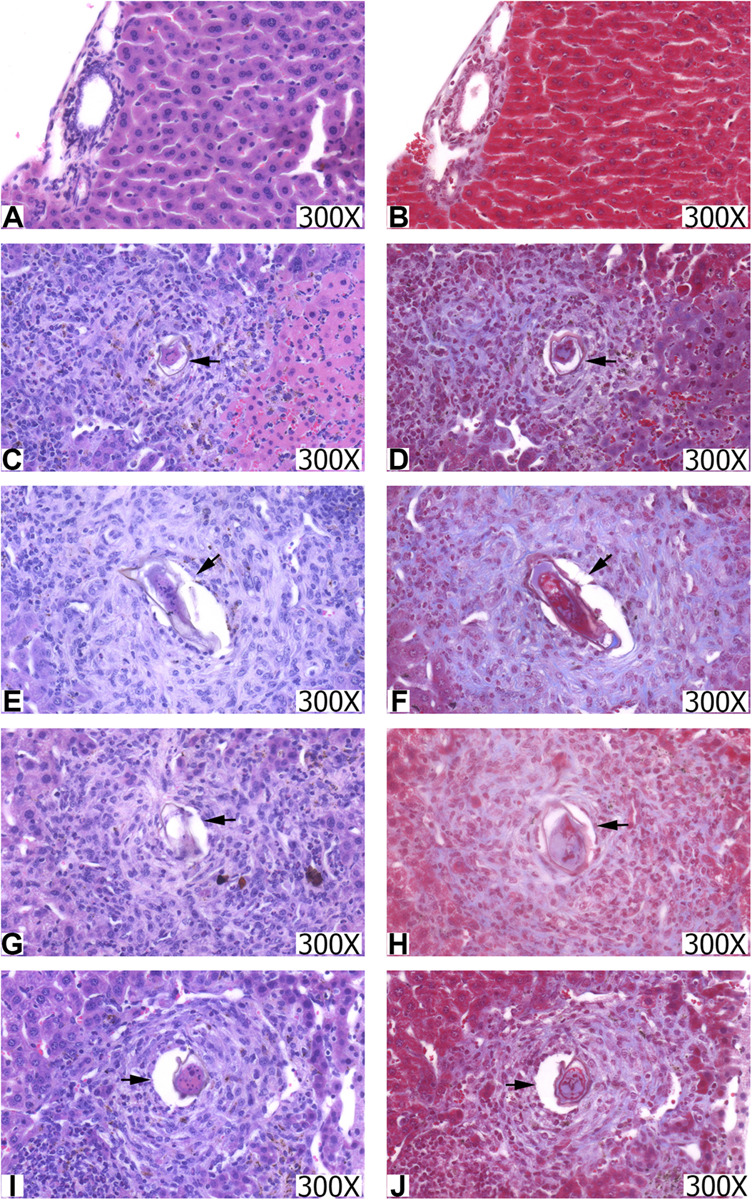
Histopathological sections of the liver from the groups CTRL **(A,B)**, INF **(C,D)**, NDPK **(E,F)**, ADSL **(G,H)**, and NDPK + ADSL **(I,J)**. The images on the left were stained with hematoxylin-eosin (HE) and those on the right with Masson trichrome (TM). Increase: 300X. The arrows indicate the presence of the granuloma around the egg.

Mice livers were analyzed through histology using HE staining to assess the presence and quantification of granulomas (24) and the appearance of cell infiltrate, and Masson trichrome (MT) to assess the collagen deposit (Figure 6). The animals in the CTRL group had well-structured and preserved liver tissue (Figure 6A). On the other hand, it was possible to observe the formation of periovular granulomas formed by lymphocytes, eosinophils, neutrophils, and epithelioid cells in all groups infected with *S. mansoni*, immunized or not (Figures 6C,E,G,I). The tissue showed structures preserved in places where there was no egg deposition.

The hepatic tissue of the CTRL group (Figure 6B) presented thin pericanalicular and perivascular collagen. The livers of all groups that were infected with *S. mansoni*, immunized or not, in addition to the presence of pericanalicular and perivascular collagen, it was possible to observe the formation of collagenous material around the eggs, along with granulomas (Figures 6B,D,F,H,J).

Table 1 shows that egg counts in the liver of animals in the NDPK and ADSL groups had a significant reduction when compared to the INF group, promoting a reduction of 49.01% and 43.48%, respectively, indicating a greater reduction in the group immunized with the recombinant enzyme NDPK. Regarding the counts of granulomas in the liver tissue, the reductions of the NDPK and ADSL groups in relation to the INF group reached 45.83% and 32.55%, respectively. Clearly, immunization with the recombinant enzyme NDPK was responsible for the greatest efficiency in reducing liver tissue granulomas in animals with experimental mansonic schistosomiasis.

**TABLE 1:** Counting of granulomas and eggs in the liver and intestine of the animals on the 48th day after infection.

	**INF**	**NDPK**	**ADSL**	**NDPK+ADSL**
Granulomas in the liver/slide	32.00 ± 5,63	17.33 ± 4.56**	21.58 ± 5.28*	24.58 ± 10.66
Reduction (%)	–	45.83%	32.55%	23.18%
Eggs in the liver/slide	21.08 ± 6.17	10.75 ± 4.01**	11.92 ± 4.83*	17.58 ± 8.87
Reduction (%)	–	49.01%	43.48%	16,60%
Eggs in the intestines/slide	18.50 ± 13.80	11.50 ± 8.58	9.17 ± 4.77	8.67 ± 4.72
Reduction (%)	–	38.84%	50.45%	53.15%

The data represent the mean ± SD (*n* = 7 animals) of two independent experiments using the parametric One-Way ANOVA test and Tukey post-test. (*) represents a statistically significant difference between the results of the experimental groups when compared with the INF group; * *p* < 0.05 and ** *p* < 0.01.

In the evaluation of egg count in mesenteric tissue, the group with the highest rate of egg reduction in the intestine was NDPK + ADSL, with 53.15%, followed by the ADSL groups, with 50.45%, and NDPK, with 38.84%. Although there is no statistical difference, immunizations with recombinant enzymes (NDPK and ADSL) were shown to be important in reducing the amount of *S. mansoni* eggs in the animals’ intestines.

## Discussion

Schistosomiasis affects almost 240 million people worldwide, and more than 700 million people live in endemic areas. The infection is prevalent in tropical and sub-tropical areas, in poor communities without potable water and adequate sanitation (25).

The morbidity triggered by this disease is related to the pathology caused by the host’s own granulomatous immune response. Due to the retention of eggs in the liver, intestine and spleen, there is the occurrence of fibrosis and organ calcification, in addition to hepatosplenomegaly (26, 27). There are still no vaccines available for schistosomiasis and there are few potential vaccine agents that have advanced to clinical tests (Sm-TSP-2, Sm-p80, and Sm14) (9). Thus, studies that seek to identify new immunogens for this disease, such as the recombinant enzymes NDPK and ADSL of *S. mansoni*, remain an urgent need for the development of a vaccine formulation.

A vaccine against schistosomiasis does not necessarily need to have sterilizing immunity, as long as it acts by limiting the parasitic burden and/or the maturation of the worms. This last attribute is important to induce a reduction in the fertility of females and, consequently, in reducing the release of eggs, which is mainly responsible for the disease morbidity (28–31).

In our study, after three immunizations with the recombinant enzymes of *S. mansoni* NDPK and ADSL, it was not possible to observe a significant reduction in the number of adult worms or in the number of eggs present in the feces and intestine. Immunization with both enzymes associated (NDPK + ADSL), has not been shown to reduce the number of adult worms or eggs in the stool. Furthermore, there was no significant reduction in the number of eggs in the liver (16.60%) and intestines (53.15%) and in granulomas in the liver (23.18%). One possible explanation is that the concentration of each recombinant enzyme used in the NDPK + ADSL group (50 μg) is half the concentration used in the groups immunized with each enzyme individually (100 μg), which may not have been sufficient to induce a significant protective effect in the analyzed parameters.

The parasite’s eggs, when established in the liver, lead to the recruitment of various inflammatory cells to the injury site, such as eosinophils, neutrophils and macrophages, to form the granuloma (32). The granulomatous response around eggs trapped in the liver tissue is initially orchestrated by CD4 + T lymphocytes, but CD8 + T cells, B cells, and macrophages have also been shown to be important in this formation. In addition to these cells, the eosinophil proved to be the main constituent of granuloma (33). Eosinophils are known for their functions as effector cells against helminth infections, although there are still discussions about their exact function (34, 35). To act on the sites of inflammation/infection, eosinophils are recruited to these sites, which can contribute to the decline of circulating eosinophils (36). In our results, there was an increase in eosinophils in the PCL of the infected animals (INF, NDPK, ADSL, and NDPK + ADSL) when compared to the CTRL group. There was no reduction in the number of eosinophils in both peripheral blood and PCL in the NDPK, ADSL, and NDPK + ADSL groups in relation to the CTRL and INF groups. On the other hand, it is noteworthy that, although there is no significant difference, the immunized groups have less eosinophils compared to INF, which may be an indication of an onset of eosinophilia modulation.

Additionally, in our results it is possible to observe that the animals’ immunization with the NDPK and ADSL enzyme within 48 days after infection showed a decrease in the percentage of granulomas in the liver and the number of eggs in the liver, when compared to the infected/untreated group, showing higher percentages of reduction to NDPK. The decrease in the number of eggs is very important, once granulomas are caused mainly by immune responses against soluble egg antigens (SEAs) (37), and even a smaller number of eggs being deposited in the tissues can lead to a reduction in the process granulomatous (38), consequently considering a possible decrease in the morbidity of this pathogenesis. Our findings are in accordance with the study by Neris et al. (39), where the authors observed that PNP and HGPRT, enzymes of the metabolic pathways of nucleotides, were able to modulate the infection by reducing the parasitic load on the liver, intestine and feces from animals infected with *S. mansoni* after 48 days of infection. Immunization with the union of NDPK and ADSL did not show a significant decrease in the reduction of granulomas, eggs in the liver or in the intestines when compared to the immunized group, suggesting that other factors may be interfering in the control of mansonic schistosomiasis.

The results obtained suggest that immunizations with recombinant enzymes evaluated individually, mainly NDPK, may be acting in the regulation of the host’s immune response against the parasite and its immunopathology associated with the development of granuloma. Immunization with the Smteg recombinant integument protein also induced a decrease in the egg count in the animals’ liver, with the rate being of 65% (40). Other recombinant proteins of *S. mansoni* also showed a reduction in the number of eggs (immunization by SmRho) and in the formation of granuloma (immunization by rP22) (41, 42).

The data presented here for NDPK seem promising, but it is worth noting that the immunological interactions necessary to eradicate invasive parasites are extremely complex and require components of both the humoral and cell-mediated immune mechanisms (43). A study with knockout mice for B cells showed the fundamental role of antibodies in inducing resistance to schistosomiasis (44). In this way, the different functional properties of antibodies make them interesting to study as they could provide important information about the progression of the disease and the effectiveness of vaccination.

In our work, the response of antibodies in the host after immunizations with the recombinant enzymes NDPK and ADSL, showed a significant increase in the production of IgG1 antibody in the groups that were previously immunized (NDPK, ADSL, and NDPK + ADSL) when compared to the CTRL and INF group, subject to sensitization of the respective enzyme. As for the IgG2a concentration, the ADSL to NDPK + ADSL groups showed increased levels when compared to the INF group. Thus, since there was an increase in the production of antibodies, both IgG1 and IgG2a, we can infer that recombinant enzymes were capable to induce specific immunity in animals against antigens of the parasite. In addition, we can observe that the concentration of antibodies of type IgG1 is higher than the concentration of antibodies of type IgG2a. The predominance of plasma IgG1 levels over IgG2a indicates that the immune response present in animals is a Th2 pattern, which is observed in the chronic phase of the disease, largely due to the presence of parasite eggs in the tissue (45, 46). The decrease in the Th2 response results in tissue damage and host mortality due to the Th1-like pro-inflammatory response. Thus, the Th2 response also acts as a protective function in the host, which is extremely important, since its appropriate regulation minimizes the damage caused by the pathology (26). A study with immunizations of the recombinant proteins Sm29 and TSP also showed an increase in IgG1, in addition to IgG3, indicating the role of these immunoglobulins in acting in the elimination of the parasite and eggs, in addition of stimulating the immune system to produce antibodies against them (47, 48). The alleged resistance to reinfection is also seen in other studies using recombinant proteins such as SmStoLP-2 (49) and Sm14-FABP (50).

Helminth infection induces a Th2 response in the host characterized by high synthesis of IgE and eosinophilia. *S. mansoni* represents a particularly potent inducer of this type of immune response, resulting in a disease characterized by high levels of IgE, IgG1, and IgG4 (51). In the Th2 type response, during the chronic phase of the disease, IgE works largely through its ability to bind to eosinophils and mast cells, both important in the response to tissue damage (52). Numerous studies of human schistosomiasis show that levels of antiparasitic IgE are related to resistance to reinfection (53–55).

In our work, a significant increase in IgE levels in responses to NDPK protein can be observed in the NDPK and NDPK + ADSL groups, in addition to an increase in IgE in response to NDPK + ADSL in all immunized groups. No increase in IgE was observed in response to the ADSL protein. Similar to our analysis, other studies have also sought to induce an increase in the protective immune response through increased production of IgE by the host, as is the case of the study conducted using Paramiosin (54) and cysteine protease cathepsin B1 (SmCB1) (56) as targets, resulting in increased protection against reinfection.

In an infection with *S. mansoni*, helper T cells are divided into two subsets. The cells of the first subset, Th1, produce IFN-γ and preferentially promote the cell-mediated immune response provided by the activation of macrophages; meanwhile, the cells of the second subset, Th2, produce interleukins IL-4 and IL-5, which promote the production of IgE and the production and activation of eosinophils, respectively, (57, 58). In the work carried out by Henri et al. (59), the authors showed that low IFN-γ production is associated with severe periportal fibrosis, indicating that the decrease in this cytokine increases the severity of the disease. In our study, we observe that there were no statistically significant changes in the measurement of the IFN-γ cytokine.

When it comes to the Th2 response, one of the main cytokines involved in regulating the response is IL-4. This cytokine has been shown to be the main regulatory molecule in Th2 cell differentiation and in the cytokine response of this response pattern. In addition, the protective function of IL-4 in this response pattern is also notable (60, 61). In fact, the Th2 response is essential for the host’s survival against *S. mansoni*. Brunet et al. (62) observed that IL-4 deficient mice had an impaired Th2 response and died earlier due to massive intestinal inflammation. When we analyzed the concentration of IL-4 in the plasma of animals in our experimental groups, we could see that there was no difference between groups when compared to INF. This stimulus is consistent with the results of eosinophils, which also did not show any difference between groups.

Our results indicate that the recombinant proteins NDPK and ADSL from the purine salvage pathway of *S. mansoni* have the potential for a possible formulation of a vaccine against mansonic schistosomiasis. Further studies are still needed to better understand the role of these proteins during the host’s immune response and how the enzymes NDPK and ADSL are acting to modulate the immune response in order to promote control and induce protection in the host against the parasite. Furthermore, we conclude that the protein with the greatest immunogenic potential for further studies is NDPK in its simple formulation.

## Data Availability Statement

All datasets presented in this study are included in the article/supplementary material.

## Ethics Statement

The animal study was reviewed and approved by Ethics Committee on the Use of Animals (CEUA) of the Federal University of São Carlos – UFSCar, under the protocol no. 2-022/2014.

## Author Contributions

DN, AF, RC, TC, LR, JT, BF, and TM participated in the performance of assays. CC, CN, and YA participated in data analysis. SA, ES, and HP were responsible for materials acquisition, analysis, or interpretation of data. FA participated in the production of the manuscript, acquisition of funding, and coordination of the project. All authors contributed to writing or critical review of the work for intellectual content and approved the final version.

## Conflict of Interest

The authors declare that the research was conducted in the absence of any commercial or financial relationships that could be construed as a potential conflict of interest.
